# Sun Exposure, Vitamin D Receptor Genetic Variants, and Risk of Breast Cancer in the Agricultural Health Study

**DOI:** 10.1289/ehp.1206274

**Published:** 2013-11-19

**Authors:** Lawrence S. Engel, Jaya Satagopan, Camelia S. Sima, Irene Orlow, Urvi Mujumdar, Joseph Coble, Pampa Roy, Sarah Yoo, Dale P. Sandler, Michael C. Alavanja

**Affiliations:** 1 Department of Epidemiology, University of North Carolina Gillings School of Global Public Health, Chapel Hill, North Carolina, USA; 2 Department of Epidemiology and Biostatistics, Memorial Sloan-Kettering Cancer Center, New York, New York, USA; 3 Division of Cancer Epidemiology and Genetics, National Cancer Institute, National Institutes of Health, Department of Health and Human Services, Bethesda, Maryland, USA; 4 National Institute of Environmental Health Sciences, National Institutes of Health, Department of Health and Human Services, Research Triangle Park, North Carolina, USA

## Abstract

Background: Epidemiologic evidence suggests a negative relation between sunlight exposure and breast cancer risk. The hypothesized mechanism is sunlight-induced cutaneous synthesis of vitamin D.

Objectives: Our goal was to examine sun exposure and its interaction with vitamin D receptor (*VDR)* gene variants on breast cancer risk.

Methods: We examined sun exposure and breast cancer incidence among 31,021 private pesticide applicators’ wives, including 578 cases, enrolled in the prospective Agricultural Health Study cohort and followed 8.6 years on average. We estimated interactions between sun exposure, *VDR* variants, and breast cancer in a nested case–control study comprising 293 cases and 586 matched controls. Information on sun exposure was obtained by questionnaire at cohort enrollment. Relative risks were estimated using Cox proportional hazards regression for the cohort data and conditional logistic regression for the nested case–control data.

Results: We observed a small decrease in breast cancer risk in association with usual sun exposure of ≥ 1 hr/day (versus < 1 hr/day) 10 years before the start of follow-up among all participants [hazard ratio (HR) = 0.8; 95% CI: 0.6, 1.0]. The association appeared to be slightly stronger in relation to estrogen receptor–positive tumors (HR = 0.7; 95% CI: 0.5, 0.9) than estrogen receptor–negative tumors (HR = 1.1; 95% CI: 0.6, 2.1). The HR for joint exposure ≥ 1 hr/day of sunlight and one *VDR* haplotype was less than expected given negative HRs for each individual exposure (interaction *p*-value = 0.07).

Conclusion: Our results suggest that sun exposure may be associated with reduced risk of breast cancer, but we did not find clear evidence of modification by *VDR* variants. Larger studies are warranted, particularly among populations in whom low levels of usual sun exposure can be more precisely characterized.

Citation: Engel LS, Satagopan J, Sima CS, Orlow I, Mujumdar U, Coble J, Roy P, Yoo S, Sandler DP, Alavanja MC. 2014. Sun exposure, vitamin D receptor genetic variants, and risk of breast cancer in the Agricultural Health Study. Environ Health Perspect 122:165–171; http://dx.doi.org/10.1289/ehp.1206274

## Background

Epidemiologic studies have reported a negative relation between sunlight exposure and risk of breast cancer. The hypothesized mechanism for this relationship is sunlight (ultraviolet B)–induced dermal synthesis of vitamin D, which experimental and nonexperimental evidence suggests may reduce risk of several cancers, including breast cancer. Dermal synthesis is the primary source of vitamin D for most individuals, with diet and supplements generally being minor contributors (Holick 2007). In dermal synthesis, 7-dehydrocholesterol in the skin is converted to vitamin D_3_, which is then hydroxylated in the liver to the prohormone 25-hydroxy vitamin D [25(OH)D], the principal circulating form of vitamin D. 25(OH)D is converted primarily in the kidneys to 1,25-dihydroxyvitamin D [1,25(OH)_2_D], the biologically active form of vitamin D, which exerts a range of anticarcinogenic effects (Holick 2007; Krishnan and Feldman 2011). Thus, sunlight and other factors that affect circulating levels of 25(OH)D may influence cancer risk.

Most cohort and case–control studies that have examined sunlight and/or ultraviolet light (UV) exposure—either through self-reported personal behaviors or via ambient levels at place of residence—have reported evidence of a negative association with breast cancer (Anderson et al. 2011b; Engel et al. 2011; John et al. 1999, 2007; Knight et al. 2007; Millen et al. 2009; Yang et al. 2011). In addition, several ecologic studies have reported negative correlations between measures of sunlight exposure (based on latitude, regional monitoring data, or acid haze) and breast cancer incidence (Grant 2010; Mohr et al. 2008) or mortality (Garland et al. 1990; Gorham et al. 1989; Grant 2002, 2010). Results from studies of serum 25(OH)D levels and breast cancer risk have been inconsistent, which may be due to differences in the timing of serum 25(OH)D measurement relative to cancer diagnosis (Gandini et al. 2011); the inadequacy of a single blood sample in many populations for assessing individuals’ usual circulating 25(OH)D levels, which vary by season and possibly over years (Rejnmark et al. 2006); or to the possibility of false-positive findings in some studies.

1,25(OH)_2_D exerts most of its known physiological effects through binding to the vitamin D receptor (VDR) (Krishnan and Feldman 2011). The VDR, which is expressed in normal breast tissue and most breast tumors (Welsh et al. 2003), regulates transcription of genes involved in cellular growth, differentiation, apoptosis, angiogenesis, and metastasis (Guyton et al. 2003; Krishnan and Feldman 2011; Lowe et al. 2003). Experimental studies on mammary tumor cell lines from *VDR*-knockout mice have shown that VDR is necessary for 1,25(OH)_2_D to induce cell cycle arrest and apoptosis in breast cancer cells (Zinser et al. 2003). In addition, the susceptibility of breast and other tissues to tumorigenesis was reported to be increased in VDR-deficient mice (Bouillon et al. 2008). The VDR is encoded by a large gene containing 14 exons that span approximately 75 kb (Crofts et al. 1998; Miyamoto et al. 1997).

In the present study we investigated the risk of breast cancer in relation to sun exposure and its interaction with *VDR* gene variants among wives of farmers in a large, prospective, two-state agricultural cohort. This population has a very wide range of sun exposure; detailed, prospective data on demographic, lifestyle, and occupational factors; and a high rate of follow-up. This research was motivated in part by previous reports of reduced risks of breast cancer among female farmers and agricultural workers relative to the general population (Duell et al. 2000; Fleming et al. 1999; Pukkala and Notkola 1997; Wiklund and Dich 1994).

## Methods

*Study population*. Participants were wives of private pesticide applicators, mainly farmers, from Iowa and North Carolina who enrolled in the prospective Agricultural Health Study (AHS) cohort between 1993 and 1997 (Alavanja et al. 1996). A total of 32,127 wives (75% of those eligible) enrolled in the cohort via self-administered questionnaire. Cancer cases were identified through population-based cancer registries in Iowa and North Carolina, and vital status was ascertained through state death registries and the National Death Index (http://www.cdc.gov/nchs/ndi.htm). Estrogen receptor (ER) and progesterone receptor (PR) status of the tumor was available from the registries for 475 (82.2%) and 447 (77.3%) cases, respectively. Excluding 1,106 wives with a malignant cancer diagnosis other than nonmelanoma skin cancer before enrollment left 31,021 participants for the present cohort analyses. Among these, 578 were diagnosed with malignant breast cancer [*International Classification of Diseases for Oncology, 2nd edition* (Percy et al. 1990), codes C50.0–C50.9] between enrollment and 31 December 2004. In addition, 23,676 wives (74% of those enrolled) completed a follow-up telephone interview approximately 5 years after enrollment, at which time approximately 60% provided a mouthwash rinse sample for extraction of buccal cell DNA. More than 98% of the wives in this cohort were white, and 99% were non-Hispanic. The nested case–control study included 293 incident breast cancer cases with a mouthwash sample (50.7% of eligible cases) and two controls with mouthwash samples who were randomly matched with replacement to each case by race (white, Hispanic and non-Hispanic; other), state (Iowa, North Carolina), age at enrollment (5-year age groups), and enrollment period (1993–1995, 1996–1997). In addition, on the diagnosis date of a given case, eligible controls also had to be alive, have no cancer diagnoses, and be living in the same state as the case. A total of 879 cases and controls were selected. Because controls were selected with replacement, which provides an unbiased sample from the cohort (Rothman and Greenland 1998), 19 participants were each selected as controls for 2 cases, and 4 participants were each selected as both a control and, at a later time point, a case. Cohort members who returned a mouthwash sample were similar to those who did not with regard to a range of demographic, lifestyle, and medical factors, suggesting that selection bias related to provision of a biospecimen is unlikely to substantially influence estimated associations (Engel et al. 2002). Only 263 women (0.8% of all participants) were lost to follow-up. The average follow-up duration was 8.6 years. Participants provided informed consent for the AHS. The institutional review boards of the National Institutes of Health and its contractors approved the AHS. The institutional review boards of Memorial Sloan-Kettering Cancer Center and the University of North Carolina at Chapel Hill approved the present study.

*Exposure assessment*. All sun exposure information was obtained at cohort enrollment. Questions included *a*) “In the growing season, how many hours a day do you generally spend in the sun?” at enrollment and also 10 years before enrollment, with choices of < 1, 1–2, 3–5, 6–10, > 10 hr/day, and *b*) “In the growing season when you work in the sun, what type(s) of sun protection do you usually use?” with choices of sunscreen/sunblock, baseball-type cap, other kind of hat with brim, long-sleeved shirt, or none of the above. The questionnaires also elicited information on a range of demographic, lifestyle, health, agricultural, and reproductive factors. Prediagnostic data on menopausal status and age at menopause were also obtained from 5-year follow-up interviews. (Questionnaires are available at http://www.aghealth.org.)

*Genotyping in the nested case–control study*. Twenty-six single nucleotide polymorphisms (SNPs) in *VDR* [rs2544038, rs739837, rs731236 (TaqI), rs2239182, rs2107301, rs2239181, rs2238139, rs2189480, rs3782905, rs7974708, rs11168275, rs2408876, rs1989969, rs2238135, rs10875694, rs3922882, rs11168287, rs7299460, rs11168314, rs4073729, rs3923693, rs4760674, rs6823, rs2071358, rs7975232 (ApaI), rs2228570 (FokI)] were genotyped, as described in our related work (Engel et al. 2012). The *VDR* haplotype structure of our study population was comparable to that observed among whites by Nejentsev et al. (2004), so linkage disequilibrium blocks were defined using the naming convention of Nejentsev et al. (2004).

*Data analysis*. We estimated associations between breast cancer and usual sun exposure, both at enrollment and 10 years before enrollment, using the five exposure categories specified in the questionnaire. For the nested case–control analyses, the upper two categories were combined because of small sample numbers. The lowest category (< 1 hr/day) was used as the reference category in all analyses. The majority of women (84.5%) reported the same levels of sun exposure during the two time periods. Therefore, we estimated associations according to time period using separate models and did not create a composite exposure estimate. We also estimated the association between breast cancer and sun exposure for ≥ 1 hr/day compared with < 1 hr/day.

Cohort study of sun exposure. We used Cox proportional hazards regression to calculate hazard ratios (HRs) and 95% confidence intervals (CIs) for the association between breast cancer and each measure of sunlight exposure. Person-years at risk for each participant were calculated from date of enrollment until the earliest of the following: first breast cancer diagnosis, first malignant non-breast cancer diagnosis (excluding nonmelanoma skin cancer), movement out of state, death, or 31 December 2004. All analyses were adjusted for known breast cancer risk factors, including age (< 40, 40–49, 50–59, 60–69, ≥ 70 years), race (white, Hispanic and non-Hispanic; other), age at menopause (premenopausal, < 45, 45–49, 50–54, ≥ 55 years, with status allowed to change during follow-up, based on self-reported age at which participant had her last menstrual period), and first-degree family history of breast cancer (yes, no). Analyses were additionally adjusted for state (Iowa, North Carolina) and for combined parity and age at first birth (1 birth, by age 30 years; ≥ 2 births, first by age 30 years; nulliparous or all births after age 30 years), with nulliparous women and those with first births after age 30 years combined because of the small number of nulliparous cases (*n* = 6). Body mass index, age at menarche, smoking status, and education were not included in the final models because they did not change risk estimates by at least 10%. Time-varying covariates (menopausal status, age at menopause) were classified at each time point based on the most recent value reported; only values reported before the end of follow-up for each participant were used.

We also performed analyses stratified by ER/PR status, menopausal status at diagnosis, family history of breast cancer, and usual use of sunscreen. We included only women with non-missing data for a given stratification factor, including all noncases in each analysis by ER/PR status and menopausal status.

Nested case–control study of sun exposure and gene–environment interaction. Conditional logistic regression was used to estimate odds ratios (ORs) and 95% CIs for usual sun exposure and interactions with genetic variants. Because the genotype data were unphased, we estimated expected haplotypes and their frequencies using the haplo.stats software (Sinnwell and Schaid 2013) in R, version 2.3 (R Foundation for Statistical Computing, Vienna, Austria). We used these as independent variables in regression models (Kraft et al. 2005) together with sun exposure (< 1 hr/day, ≥ 1 hr/day) and a product term. We examined only the most common 50% of haplotypes in each linkage disequilibrium block, comprising six haplotypes in block B and seven haplotypes in block C (Engel et al. 2012) (see Supplemental Material, Table S2). We present interaction results in the tables only for SNPs and haplotypes that either had significant main effects in univariate analyses (*p* < 0.05) or showed evidence of departure from multiplicativity in interaction analyses. The multiplicative interaction between sun exposure and each SNP was evaluated via the statistical significance (*p* < 0.05) of the likelihood ratio test comparing the models with and without the product term. All analyses were adjusted for age at menopause, combined parity and age at first birth, and first-degree family history of breast cancer, as described above, based on status at enrollment.

There was insufficient DNA for genotyping for 23 cases and 32 controls. To account for missing genotype and sun exposure data, we used the missing-indicator method (Huberman and Langholz 1999) in our analysis of gene–sun interactions. This method allows all participants to be included in analyses and maintains case–control matching, and produces an OR estimate that is a compromise (i.e., a weighted average) between the estimates from a matched analysis of complete sets and an unmatched analysis of incomplete sets. Analysis confirmed lack of heterogeneity in ORs between complete sets and incomplete sets, which is necessary for the validity of this method (Huberman and Langholz 1999).

In both the cohort and the case–control analyses, missing data for adjustment covariates were imputed using IVEware (Institute for Social Research, University of Michigan, Ann Arbor, MI). This program, which assumes an ignorable-missing-data mechanism, simultaneously imputes values for specified variables by fitting a sequence of regression models and drawing values from the corresponding predictive distributions. Missing values were imputed for race (3.2%), family history of breast cancer (5.0%), parity (17.6%), and age at menopause (2.0%). Risk estimates including imputed data were not materially different from those including only observed data, so we present risk estimates adjusted using the imputed and observed data.

All statistical analyses were performed using SAS, version 9.1 (SAS Institute Inc., Cary, NC), except where otherwise noted. Statistical significance was assessed at the 5% level. Tests for trend were assessed using midpoints of categories as continuous measures. In analyses of covariate risk (Table 1; see also Supplemental Material, Table S1), all covariates were adjusted for the other covariates, except where indicated, and with no imputed data. We did not adjust *p*-values for multiple comparisons because of the exploratory nature of our genetic analyses. Analyses were based on AHS data releases P1REL0506.01 and P2REL0506.04.

**Table 1:** Selected characteristics at enrollment of wives in the AHS cohort [cases (*n* = 578), noncases (*n* = 30,443)].

Characteristic	Cases [*n* (%)]	Non­cases [*n* (%)].	Adjusted HR^*a*^**(95% CI)
Age (years)
18–39	55 (9.5)	9,747 (32.0)	1 (Reference)
40–49	141 (24.4)	8,775 (28.8)	2.8 (2.1, 3.9)
50–59	214 (37.0)	7,025 (23.1)	5.6 (4.0, 8.0)
60–69	127 (22.0)	3,938 (12.9)	6.2 (4.2, 9.2)
70–86	41 (7.1)	958 (3.2)	8.6 (5.4, 13.7)
Race
White (Hispanic and non-Hispanic)	547 (98.2)	28,962 (98.2)	1 (Reference)
Other	10 (1.8)	517 (1.8)	0.9 (0.5, 1.8)
Missing	21	964
State of residence
Iowa	362 (62.6)	20,469 (67.2)	1 (Reference)
North Carolina	216 (37.4)	9,974 (32.8)	1.1 (0.9, 1.3)
Highest educational level
< High school	30 (6.0)	1,435 (5.4)	0.7 (0.5, 1.1)
High school	223 (44.2)	10,604 (40.1)	0.9 (0.8, 1.1)
> High school	251 (49.8)	14,412 (54.5)	1 (Reference)
Missing	74	3,992
Smoking
Never	394 (73.1)	20,772 (72.4)	1 (Reference)
Former	105 (19.5)	4,925 (17.2)	1.2 (0.9, 1.7)
Current	40 (7.4)	3,003 (10.5)	1.3 (0.9, 1.9)
Missing	39	1,743
First-degree family history of breast cancer
Yes	114 (20.9)	3,289 (11.4)	1.8 (1.4, 2.2)
No	431 (79.1)	25,643 (88.6)	1 (Reference)
Missing	33	1,511
Body mass index (kg/m^2^)
< 25.0	191 (44.6)	10,417 (50.6)	1 (Reference)
25.0–29.9	143 (33.4)	6,472 (31.4)	1.0 (0.8, 1.2)
≥ 30.0	94 (22.0)	3,709 (18.0)	1.2 (1.0, 1.6)
Missing	150	9,845
Age at menarche (years)
< 12	54 (13.4)	2,790 (15.4)	1 (Reference)
12–14	315 (78.0)	13,710 (75.5)	0.9 (0.7, 1.2)
≥ 15	35 (8.7)	1,656 (9.1)	1.0 (0.7, 1.4)
Missing	174	12,287
Parity
Nulliparous	6 (1.2)	621 (2.5)	0.9 (0.4, 2.0)
1	54 (10.7)	2,359 (9.4)	1.2 (0.9, 1.7)
≥ 2	444 (88.1)	22,075 (88.1)	1 (Reference)
Missing	74	5,388
Age at first birth (years)^*b*^
≤ 20	91 (24.8)	3,889 (23.4)	1 (Reference)
20–30	239 (65.1)	11,700 (70.3)	1.0 (0.7, 1.2)
> 30	37 (10.1)	1,044 (6.3)	1.8 (1.2, 2.6)
Missing	131	7,801
Menopausal status
Post­menopausal	325 (64.5)	11,054 (43.0)	0.9 (0.7, 1.1)
Pre­menopausal	179 (35.5)	14,663 (57.0)	1 (Reference)
Missing	74	4,726
Age at menopause (years)^*c*^
< 45	102 (32.2)	4,249 (39.2)	1 (Reference)
45–49	81 (25.6)	2,659 (24.5)	1.0 (0.7, 1.3)
50–54	107 (33.8)	3,085 (28.5)	1.1 (0.8, 1.4)
≥ 55	27 (8.5)	846 (7.8)	1.0 (0.6, 1.5)
Missing	8	215
Usual sunblock use at enrollment
Yes	242 (41.9)	12,943 (42.5)	1.0 (0.8, 1.2)
No	336 (58.1)	17,500 (57.5)	1 (Reference)
Tumor ER status
ER^+^	315 (75.0)	NA
ER^–^	105 (25.0)	NA
Missing	158
Tumor PR status
PR^+^	280 (67.3)	NA
PR^–^	136 (32.7)	NA
Missing	162
NA, not applicable. ^***a***^HRs were estimated using Cox proportional hazards regression, with all factors adjusted for the other factors in the table, except where indicated, and with no imputed data. ^***b***^Restricted to parous women, with no imputed data. ­^***c***^Restricted to post­menopausal women, with no imputed data.			

## Results

Selected characteristics of the women in the cohort and in the nested case–control study are provided in Table 1 (see also Supplemental Material, Table S1). Most of the women in the cohort (60.3%) were under 50 years of age at enrollment, although, as expected, cases were on average older than noncases/controls. Over 67% of the participants lived in Iowa. Almost all of the women (97.5%) had had at least one birth and about 43% were postmenopausal at enrollment. Distributions of most demographic and lifestyle factors were similar for the 578 cases in the cohort (Table 1) and the 293 cases included in the nested case–control study (see Supplemental Material, Table S1). However, a slightly larger proportion of cases in the nested case–control study than in the cohort were from Iowa (66.9% vs. 62.6%) and had education beyond high school (54.8% vs. 49.8%). Approximately 98% of both the cohort and the case–control sample were white.

*Sun exposure*. The range of sun exposure during the growing season among study participants was very wide (Table 2). Usual sun exposure of ≥ 6 hr/day was reported by 12.4% of participants who provided information on sun exposure for the period around enrollment and by 21.8% of those who provided information for the period 10 years before enrollment, whereas 27.0% and 17.9% reported < 1 hr/day for each of these periods, respectively. Sun exposure data were missing for 29–33% of the participants.

**Table 2:** Sunlight exposure and breast cancer risk among wives in the cohort [cases (*n* = 578), noncases (*n* = 30,443)] and in the nested case–control study [cases (*n* = 293), noncases (*n* = 586)].

Characteristic	Cohort			Nested case–­control study		
	Cases [*n* (%)]	Non­cases [*n* (%)]	Adjusted HR^*a*^ (95% CI)	Cases [*n* (%)]	Controls [*n* (%)]	Adjusted OR^*b*^ (95% CI)
Usual hours of sun exposure per day at enrollment
< 1 hr	139 (30.8)	5,804 (26.9)	1 (Reference)	78 (31.5)	142 (29.9)	1 (Reference)
1–2 hr	136 (30.2)	6,962 (32.3)	0.9 (0.7, 1.1)	71 (28.6)	148 (31.2)	0.8 (0.5, 1.2)
3–5 hr	125 (27.7)	6,129 (28.4)	0.9 (0.7, 1.2)	72 (29.0)	123 (25.9)	1.0 (0.7, 1.6)
6–10 hr	43 (9.5)	2,156 (10.0)	0.9 (0.7, 1.3)	21 (8.5)	52 (10.9)	0.8^**^(0.4, 1.3)^*b*^
> 10 hr	8 (1.8)	521 (2.4)	0.7 (0.3, 1.4)	6 (2.4)	10 (2.1)
< 1 hr	139 (30.8)	5,804 (26.9)	1 (Reference)	78 (31.5)	142 (29.9)	1 (Reference)
≥ 1 hr	312 (69.2)	15,768 (73.1)	0.9 (0.7, 1.1)	170 (68.5)	333 (70.1)	0.9 (0.6, 1.2)
Missing	127	8,871		45	111
Usual hours of sun exposure per day 10 years before enrollment
< 1 hr	92 (21.7)	3,592 (17.8)	1 (Reference)	57 (24.4)	88 (19.3)	1 (Reference)
1–2 hr	103 (24.3)	5,224 (25.8)	0.8 (0.6, 1.1)	54 (23.1)	103 (22.6)	0.7 (0.5, 1.2)
3–5 hr	133 (31.4)	6,967 (34.5)	0.8 (0.6, 1.0)	74 (31.6)	154 (33.8)	0.7 (0.5, 1.1)
6–10 hr	75 (17.7)	3,569 (17.6)	0.8 (0.6, 1.1)	36 (15.4)	88 (19.3)	0.6^**^(0.4, 1.0)^*c*^
> 10 hr	20 (4.7)	869 (4.3)	0.9 (0.5, 1.4)	13 (5.6)	22 (4.8)
< 1 hr	92 (21.7)	3,592 (17.8)	1 (Reference)	57 (24.4)	88 (19.3)	1 (Reference)
≥ 1 hr	331 (78.3)	16,629 (82.2)	0.8 (0.6, 1.0)	177 (75.6)	367 (80.7)	0.7 (0.5, 1.0)
Missing	155	10,222		59	131
^***a***^HRs were estimated using Cox proportional hazards regression adjusted for age (< 40, 40–49, 50–59, 60–69, ≥ 70 years), race (white, other), state (Iowa, North Carolina), age at menopause (premenopausal, < 45, 45–49, 50–54, ≥ 55 years), combined parity and age at first birth (1 birth, by age 30 years; ≥ 2 births, first by age 30 years; nulliparous or all births after age 30 years), and first-degree family history of breast cancer (yes, no). ^***b***^ORs were estimated using conditional logistic regression, matched on age at enrollment, race, and state, and adjusted for age at menopause, combined parity and age at first birth, and first-degree family history of breast cancer, as for the cohort analyses. Missing covariate data were imputed using IVEware, a multivariate sequential regression approach. ^***c***^The upper two categories—6–10 hr and > 10 hr—were combined in nested case–­control analyses because of small sample ­numbers.						

We found little evidence of a decreasing dose–response relation between usual sun exposure and breast cancer risk for exposure either at enrollment or 10 years before enrollment (Table 2). However, we observed a small decreased risk associated with usual sun exposure of ≥ 1 hr/day compared with < 1 hr/day 10 years before enrollment (HR = 0.8; 95% CI: 0.6, 1.0) in the cohort, with similar associations for participants in Iowa (HR = 0.7; 95% CI: 0.5, 1.0) and North Carolina (HR = 0.8; 95% CI: 0.6, 1.2). At least 75% of North Carolina participants and 84% of Iowa participants resided in the same state 10 years before enrollment. As expected, patterns of association were similar between women in the cohort and women in the nested case–control study.

Negative associations with sun exposure appeared to be limited to women with no family history of breast cancer (for sun exposure ≥ 1 hr/day vs. < 1 hr/day 10 years before enrollment, HR = 0.7; 95% CI: 0.6, 0.9 and 1.2; 95% CI: 0.6, 2.1 among women without and with a family history, respectively) (Table 3). The negative association with sun exposure also appeared to be limited to women with ER^+^ tumors [for sun exposure ≥ 1 hr/day versus < 1 hr/day 10 years before enrollment, HR = 0.7; 95% CI: 0.5, 0.9 and 1.1; 95% CI: 0.6, 2.1 for ER^+^ (*n* = 315) and ER^–^ (*n* = 105) tumors, respectively]. We observed no evidence of differences in associations by menopausal status, usual use of sunscreen/sunblock, or PR status of the tumor (Table 3).

**Table 3:** HRs for sunlight exposure ≥ 1 hr/day and breast cancer risk among wives in the cohort, stratified by selected factors [cases (*n* = 578), noncases (*n* = 30,443)].

Characteristic	Cases (*n*)	Noncases (*n*)	Usual hours of sun exposure per day at enrollment [adjusted HR^*a*^ (95% CI)]	Usual hours of sun exposure per day 10 years before enrollment [adjusted HR^*a*^ (95% CI)]
Menopausal status
Premenopausal	179	14,663	1.0 (0.7, 1.4)	0.8 (0.5, 1.1)
Postmenopausal	325	11,054	0.9 (0.7, 1.1)	0.8 (0.6, 1.1)
Usual use of sunscreen/sunblock
Yes	242	12,943	0.9 (0.7, 1.1)	0.7 (0.5, 1.0)
No	336	17,500	0.9 (0.7, 1.2)	0.8 (0.6, 1.2)
Family history of breast cancer
Yes	114	3,289	1.3 (0.8, 2.1)	1.2 (0.6, 2.1)
No	431	25,643	0.8 (0.7, 1.1)	0.7 (0.6, 0.9)
Tumor ER status
ER^+^	315	NA	0.9 (0.7, 1.2)	0.7 (0.5, 0.9)
ER^–^	105	NA	0.7 (0.4, 1.1)	1.1 (0.6, 2.1)
Tumor PR status
PR^+^	280	NA	0.9 (0.7, 1.2)	0.7 (0.5, 1.0)
PR^–^	136	NA	0.7 (0.5, 1.1)	0.8 (0.5, 1.2)
^***a***^HRs were estimated using Cox proportional hazards regression adjusted for age (< 40, 40–49, 50–59, 60–69, ≥ 70 years), race (white, other), state (Iowa, North Carolina), age at menopause (premenopausal, < 45, 45–49, 50–54, ≥ 55 years), combined parity and age at first birth (1 birth, by age 30 years; ≥ 2 births, first by age 30 years; nulliparous or all births after age 30 years), and first-degree family history of breast cancer (yes, no). Factors that are being stratified on are not adjusted for in those models. Missing covariate data were imputed using IVEware, a multivariate sequential regression approach.				

Adjustment for self-reported duration of pesticide use and self-reported measures of occupational and recreational physical activity from the enrollment questionnaire did not materially alter risk estimates (data not shown). Results were also similar in subanalyses restricted to whites (data not shown), which was expected given the small proportion of nonwhites in this study.

*Gene–environment interactions*. Tables 4 and 5 present results for only the five SNPs and three haplotypes, respectively, that either had significant main effects in univariate analyses (*p* < 0.05) or showed evidence of departure from multiplicativity in interaction analyses. The interaction between rs2239181 and usual sun exposure 10 years before enrollment on breast cancer risk showed some evidence of a departure from multiplicativity (Table 4). Among those with the T/T genotype at rs2239181, usual sun exposure ≥ 1 hr/day was associated with a 30% decrease in the odds of breast cancer (OR = 0.7; 95% CI: 0.4, 1.1) relative to usual sun exposure < 1 hr/day. In contrast, among those with T/G or G/G genotypes (combined), usual sun exposure ≥ 1 hr/day was associated with only a 14% decrease in the odds of breast cancer relative to those with usual sun exposure < 1 hr/day (OR = 1.2 vs. OR = 1.4; interaction *p*-value = 0.06). We found no evidence of departure from multiplicativity between other SNPs and usual sun exposure 10 years before enrollment.

**Table 4:** Selected interactions between genetic polymorphisms and usual sun exposure 10 years before enrollment on breast cancer risk among wives in the nested case–control study [cases (*n* = 293), noncases (*n* = 586)].^*a*^

Genotype	Sun exposure (hr/day)	Cases(*n*^*b*^)	Controls(*n*^*b*^)	Adjusted OR^*c*^(95% CI)	*p*‑Value for inter­action^*d*^
rs2544038
T/T	< 1	16	26	1 (Reference)
T/T	≥ 1	45	120	0.5 (0.3, 1.1)
T/C	< 1	24	45	0.9 (0.4, 2.0)
T/C	≥ 1	84	160	0.8 (0.4, 1.6)
C/C	< 1	12	11	1.9 (0.6, 5.4)
C/C	≥ 1	31	57	0.9 (0.4, 1.9)	0.12
rs2239181
T/T	< 1	38	63	1 (Reference)
T/T	≥ 1	132	302	0.7 (0.4, 1.1)
T/G or G/G	< 1	14	18	1.4 (0.6, 3.2)
T/G or G/G	≥ 1	32	44	1.2 (0.6, 2.3)	0.06
rs11168287
A/A	< 1	11	23	1 (Reference)
A/A	≥ 1	52	87	1.2 (0.5, 2.6)
A/G	< 1	29	42	1.5 (0.6, 3.5)
A/G	≥ 1	83	175	0.9 (0.4, 2.0)
G/G	< 1	12	17	1.3 (0.5, 3.9)
G/G	≥ 1	24	71	0.7 (0.3, 1.6)	0.26
rs739837
T/T	< 1	14	31	1 (Reference)
T/T	≥ 1	56	94	1.2 (0.6, 2.5)
T/G	< 1	21	33	1.3 (0.6, 3.1)
T/G	≥ 1	75	157	1.0 (0.5, 1.9)
G/G	< 1	17	19	1.8 (0.7, 4.6)
G/G	≥ 1	33	93	0.7 (0.3, 1.5)	0.13
rs7975232
A/A	< 1	14	31	1 (Reference)
A/A	≥ 1	56	94	1.2 (0.6, 2.5)
A/C	< 1	21	33	1.3 (0.6, 3.1)
A/C	≥ 1	77	160	1.0 (0.5, 1.9)
C/C	< 1	17	19	1.8 (0.7, 4.6)
C/C	≥ 1	32	94	0.6 (0.3, 1.4)	0.10
^***a***^Table includes inter­action results only for SNPs (of 26 evaluated) that either had significant main effects in univariate analyses (*p* < 0.05) or showed evidence of departure from multiplicativity in inter­action analyses. ^***b***^Because some members of matched case–­control sets had missing information on sun exposure or geno­type, the missing-indicator method was used to retain all participants (*n*_cases_ = 293, *n*_controls_ = 586) and maintain case–­control matching (see text). ^***c***^ORs were estimated using conditional logistic regression, with matching on age at enrollment (5-year age groups), race (white, other), and state (Iowa, North Carolina), and adjusted for age at menopause (premenopausal, < 45, 45–49, 50–54, ≥ 55 years), combined parity and age at first birth (1 birth, by age 30 years; ≥ 2 births, first by age 30 years; nulliparous or all births after age 30 years), and first-degree family history of breast cancer (yes, no). Missing covariate data were imputed using IVEware, a multivariate sequential regression approach. ^***d***^Based on model assuming codominant polymorphisms and dichotomous sun exposure and a multiplicative inter­action term between them.					

**Table 5:** Selected interactions^*a*^ between haplotypes in block B^*b*^ and usual sun exposure 10 years before enrollment on breast cancer risk among wives in the nested case–control study [cases (*n* = 293), noncases (*n* = 586)].

Haplotype	Sun exposure (hr/day)	Adjusted OR^*c*^ (95% CI)	*p*‑Value for inter­action
B4: GTCATTTCCTA
Not B4	< 1	1 (Reference)
Not B4	≥ 1	0.7 (0.5, 1.1)
B4	< 1	0.6 (0.2, 1.5)
B4	≥ 1	0.3 (0.2, 0.5)	0.69
B5: TCAGCTTACTA
Not B5	< 1	1 (Reference)
Not B5	≥ 1	0.7 (0.4, 1.0)
B5	< 1	0.8 (0.4, 1.5)
B5	≥ 1	0.7 (0.5, 0.8)	0.51
B6: TCAGCTTCGCA
Not B6	< 1	1 (Reference)
Not B6	≥ 1	0.5 (0.3, 0.9)
B6	< 1	0.6 (0.4, 1.0)
B6	≥ 1	0.6 (0.5, 0.7)	0.07
^***a***^Table includes inter­action results only for haplotypes (of 13 evaluated) that either had significant main effects in univariate analyses (*p* < 0.05) or showed evidence of departure from multiplicativity in inter­action analyses. ^***b***^Blocks based on Nejentsev et al. (2004), with order of SNPs in block B as follows: rs739837, rs731236, rs7975232, rs2239182, rs2107301, rs2239181, rs2238139, rs2189480, rs3782905, rs7974708, rs11168275. ^***c***^ORs were estimated using conditional logistic regression, with matching on age at enrollment (5-year age groups), race (white, other), and state (Iowa, North Carolina), and adjusted for age at menopause (premenopausal, < 45, 45–49, 50–54, ≥ 55 years), combined parity and age at first birth (1 birth, by age 30 years; ≥ 2 births, first by age 30 years; nulliparous or all births after age 30 years), and first-degree family history of breast cancer (yes, no). Missing covariate data were imputed using IVEware, a multivariate sequential regression approach.			

There was a suggestion of sub-multiplicativity between haplotype TCAGCTTCGCA (haplotype “B6”) and usual sun exposure ≥ 1 hr/day 10 years before enrollment; however, this was not significant (interaction *p*-value = 0.07) (Table 5). Among carriers of the TCAGCTTCGCA haplotype, sun exposure was not associated with the odds of breast cancer: The ORs associated with sun exposure ≥ 1 hr/day and sun exposure < 1 hr/day were both 0.6. In contrast, among noncarriers of this haplotype, sun exposure was associated with a 50% decrease in breast cancer odds (OR = 0.5; 95% CI: 0.3, 0.9). Results did not differ substantively between subgroups defined by family history of breast cancer or use of sun protection, or in subanalyses restricted to whites (data not shown).

## Discussion

Results from this large, prospective cohort study of women living and/or working on farms suggest that sunlight exposure may be associated with a reduced risk of breast cancer, particularly for ER^+^ tumors. The timing of sun exposure may be important because exposure 10 years before start of follow-up was negatively associated with breast cancer, whereas sun exposure at the start of follow-up was not, although this difference could be due to other factors such as missing data. There was some evidence that the association between usual sun exposure and risk of breast cancer was modified by one of the 13 haplotypes evaluated.

Our findings regarding sunlight exposure and breast cancer risk are consistent with most previous studies on this topic. Negative associations have been observed in both case–control studies (Anderson et al. 2011b; Knight et al. 2007) and cohort studies (Engel et al. 2011; John et al. 1999, 2007; Millen et al. 2009; Yang et al. 2011). Measures of sunlight exposure that have been negatively associated with breast cancer include self-reported time spent outdoors in daylight (Anderson et al. 2011b; John et al. 1999; Knight et al. 2007; Millen et al. 2009) and cumulative sun exposure estimates based on reflectometric measurement of skin pigmentation (John et al. 2007). Extent of sun-seeking vacations and solarium use were negatively associated with breast cancer risk in a cohort of 42,559 Swedish women followed for an average of 14.9 years (Yang et al. 2011) but not in a cohort of 41,811 Norwegian women followed for an average of 8.5 years (Edvardsen et al. 2011). Ecologic studies, while providing weaker evidence, also have been largely consistent in showing a negative correlation between breast cancer risk and potential UV exposure, based on average ground-level solar energy (Garland et al. 1990; Gorham et al. 1990), latitude (Grant 2010), or acid haze (Gorham et al. 1989). The reason for the observed difference in association by timing of exposure in our study is unclear. Some studies have suggested stronger associations at younger ages (Knight et al. 2007; Yang et al. 2011), which would be generally consistent with our findings, although other studies have found similar associations across age groups (Anderson et al. 2011b; John et al. 2007).

We found no evidence of a dose–response relation between self-reported sun exposure ≥ 1 hr/day and breast cancer risk, but the relative risk of breast cancer did appear to be reduced in association with ≥ 1 hr/day of usual sun exposure compared with < 1 hr/day. The exposure distribution in this occupationally exposed population is likely skewed high compared with general population samples in previous studies. However, once individuals achieve a circulating 25(OH)D level of about 40 ng/mL, the effects of additional sun/UV exposure appear to become blunted, with a much lower rate of increase in circulating 25(OH)D per unit of sun exposure (Hollis 2005). Self-reported exposure information reflecting usual behavior over a long time period may be too imprecise to measure incremental reductions in risk from longer durations of sun exposure.

We observed no modification of the association between sunlight exposure and breast cancer risk by sunscreen use, which is consistent with the lack of association between sunscreen use and breast cancer risk reported in other studies (Anderson et al. 2011b; Knight et al. 2007; Kuper et al. 2009). This may be due to the fact that many people apply insufficient sunscreen and do not reapply it as frequently as needed (Norval and Wulf 2009). Indeed, sunscreen use can be a poor predictor of 25(OH)D levels (Thieden et al. 2009).

Evidence that the relationship between vitamin D and breast cancer risk differs by hormone receptor status of the tumor is conflicting. Blackmore et al. (2008), in a population-based study of 759 cases and 1,135 controls, observed reduced risks of similar magnitude for ER^+^/PR^+^ tumors, ER^–^/PR^–^ tumors, and ER^+^/PR^–^ tumors associated with increased vitamin D intake via sun and diet. In contrast, a study involving 1,019 incident cases within the prospective Women’s Health Study (Lin et al. 2007) and another study involving 2,855 incident cases within the prospective Cancer Prevention Study II Nutrition Cohort (McCullough et al. 2005) reported stronger negative associations between dietary vitamin D intake and both ER^+^ or PR^+^ tumors. However, a study of 2,440 incident cases within the prospective Iowa Women’s Health Study observed stronger negative associations with ER^–^ or PR^–^ tumors (Robien et al. 2007). *In vitro* studies suggest that ER^+^ breast cancer cell lines are generally more sensitive than ER^–^ cell lines to 1,25(OH)_2_D–mediated growth regulation (Welsh et al. 2002).

Studies that have examined interactions between *VDR* variants and markers of vitamin D—including sun exposure, serum 25(OH)D, and dietary vitamin D intake—on breast cancer risk have produced inconsistent, but largely null, results. A case–control study of breast cancer reported limited evidence of an interaction between *BsmI* (rs1544410) genotype and serum 25(OH)D concentrations measured in blood samples collected after diagnosis (Lowe et al. 2005). A case–control study that examined seven of the same polymorphisms as the present study (rs731236, rs739837, rs1989969, rs2228570, rs7975232, rs2107301, rs2238135) found, like the present study, no significant interactions between sun exposure and these polymorphisms (Anderson et al. 2011a). However, Anderson et al. (2011a) did observe a significant interaction between dietary vitamin D intake and rs2238135. Other studies have reported no modification of associations between measured or inferred vitamin D status and breast cancer by the *VDR* polymorphisms *BsmI* (Chen et al. 2005; McCullough et al. 2007), *FokI* (Abbas et al. 2008; Chen et al. 2005; John et al. 2007; McCullough et al. 2007), *TaqI* (Abbas et al. 2008; John et al. 2007; McCullough et al. 2007), or *ApaI* (rs7975232, also evaluated in the present study) (McCullough et al. 2007).

Limitations of this study include use of self-reported usual sun exposure, which likely introduced some exposure misclassification. However, studies indicate that reliability of recall of usual or total sun exposure is good, with interclass correlation coefficients of about 0.7–0.8 (English et al. 1998; Rosso et al. 2002); that self-reported sun exposure correlates with circulating 25(OH)D levels (Hanwell et al. 2010; Sowers et al. 1986); and that reliability of reporting for a range of factors among Agricultural Health Study participants is good to excellent, with percent agreements of 50–60% for measures of pesticide use and 71–76% for amount of alcohol and tobacco use (Blair et al. 2002). The appreciable amount of missing sun exposure data may have introduced some bias in the present study. About 22% of cases and 29% of noncases were missing data on usual sun exposure at enrollment, whereas 29% of cases and 34% of noncases were missing data on usual sun exposure 10 years before enrollment. The reasons for the differences in percent missing are unclear. However, any exposure misclassification among those who provided sun exposure information in this study is likely nondifferential with regard to disease status because exposure information was collected before breast cancer diagnosis. As in any study, there may also be uncontrolled confounding, although we evaluated a wide range of potential confounders and included any that were found to affect risk estimates. Although the 5-year follow-up interview may have occurred after breast cancer diagnosis for some cases, only covariate data collected before diagnosis for the cases and their matched controls were included in this study. Also, the minimum sun exposure category in the questionnaire was up to 1 hr, and the rate of vitamin D synthesis per amount of sun exposure may decrease within this time period. Nonetheless, evidence suggests that vitamin D levels continue to rise with increasing sun exposure above levels attainable through casual exposure (Adams et al. 1982; Haddad and Chyu 1971) and our cohort had a very wide range of exposure. Although we examined only sun exposure as a vitamin D source in the present study, sun exposure accounts for the large majority of circulating 25(OH)D in most people (Holick 2007). The relatively small sample size of the nested case–control study limited our ability to estimate interactions. In addition, some observed associations may be due to chance because of the number of comparisons performed. Last, the generalizability of this study may be limited primarily to whites because of the small proportion of nonwhites in the study population and differences in vitamin D synthesis by skin color.

Strengths of this study include collection of all information on exposures and covariates before disease diagnosis; thus, any misclassification was likely nondifferential with regard to disease status. In addition, this cohort is large and includes a substantial number of incident cases. Follow-up of this cohort is excellent. Also, this cohort has an unusually wide range of usual sun exposure compared with the general population, with a substantial proportion of women in the upper end of the exposure distribution providing greater exposure contrasts, although no dose–response association was observed. Reliability of reporting for a range of lifestyle and occupational factors is good to excellent in this cohort (Blair et al. 2002). Finally, this study had detailed data at baseline and at 5 years on potential confounders and effect modifiers.

## Conclusion

Our results suggest that sun exposure may be associated with reduced risk of breast cancer, but we did not find clear evidence of modification of this association by variants in the *VDR* gene. Our results further suggest that this association may be stronger for ER^+^ tumors, although these analyses are based on a relatively small sample size. Larger studies, particularly among populations in which usual sun exposure at the low end can be more precisely characterized, are warranted to help clarify this relationship.

## Supplemental Material

(295 kB) PDF

## References

[r1] Abbas S Nieters A Linseisen J Slanger T Kropp S 2008 Vitamin D receptor gene polymorphisms and haplotypes and postmenopausal breast cancer risk. Breast Cancer Res 10 2 R31; 10.1186/bcr1994 18419802 PMC2397533

[r2] Adams JS, Clemens TL, Parrish JA, Holick MF (1982). Vitamin-D synthesis and metabolism after ultraviolet irradiation of normal and vitamin-D-deficient subjects.. N Engl J Med.

[r3] Alavanja MC, Sandler DP, McMaster SB, Zahm SH, McDonnell CJ (1996). The Agricultural Health Study.. Environ Health Perspect.

[r4] Anderson LN, Cotterchio M, Cole DE, Knight JA (2011a). Vitamin D-related genetic variants, interactions with vitamin D exposure, and breast cancer risk among Caucasian women in Ontario.. Cancer Epidemiol Biomarkers Prev.

[r5] Anderson LN, Cotterchio M, Kirsh VA, Knight JA (2011b). Ultraviolet sunlight exposure during adolescence and adulthood and breast cancer risk: a population-based case-control study among Ontario women.. Am J Epidemiol.

[r6] Blackmore KM, Lesosky M, Barnett H, Raboud JM, Vieth R (2008). Vitamin D from dietary intake and sunlight exposure and the risk of hormone-receptor-defined breast cancer.. Am J Epidemiol.

[r7] Blair A, Tarone R, Sandler D, Lynch CF, Rowland A (2002). Reliability of reporting on life-style and agricultural factors by a sample of participants in the Agricultural Health Study from Iowa.. Epidemiology.

[r8] Bouillon R, Carmeliet G, Verlinden L, van Etten E, Verstuyf A (2008). Vitamin D and human health: lessons from vitamin D receptor null mice.. Endocr Rev.

[r9] Chen WY, Bertone-Johnson ER, Hunter DJ, Willett WC, Hankinson SE (2005). Associations between polymorphisms in the vitamin D receptor and breast cancer risk.. Cancer Epidemiol Biomarkers Prev.

[r10] Crofts LA, Hancock MS, Morrison NA, Eisman JA (1998). Multiple promoters direct the tissue-specific expression of novel N-terminal variant human vitamin D receptor gene transcripts.. Proc Natl Acad Sci USA.

[r11] Duell EJ, Millikan RC, Savitz DA, Newman B, Smith JC (2000). A population-based case-control study of farming and breast cancer in North Carolina.. Epidemiology.

[r12] Edvardsen K, Veierod MB, Brustad M, Braaten T, Engelsen O (2011). Vitamin D-effective solar UV radiation, dietary vitamin D and breast cancer risk.. Int J Cancer.

[r13] Engel LS, Orlow I, Sima CS, Satagopan J, Mujumdar U (2012). Vitamin D receptor gene haplotypes and polymorphisms and risk of breast cancer: a nested case–control study.. Cancer Epidemiol Biomarkers Prev.

[r14] Engel LS, Rothman N, Knott C, Lynch CF, Logsden-Sackett N (2002). Factors associated with refusal to provide a buccal cell sample in the Agricultural Health Study.. Cancer Epidemiol Biomarkers Prev.

[r15] Engel P, Fagherazzi G, Mesrine S, Boutron-Ruault MC, Clavel-Chapelon F (2011). Joint effects of dietary vitamin D and sun exposure on breast cancer risk: results from the French E3N cohort.. Cancer Epidemiol Biomarkers Prev.

[r16] English DR, Armstrong BK, Kricker A (1998). Reproducibility of reported measurements of sun exposure in a case-control study.. Cancer Epidemiol Biomarkers Prev.

[r17] Fleming LE, Bean JA, Rudolph M, Hamilton K (1999). Cancer incidence in a cohort of licensed pesticide applicators in Florida.. J Occup Environ Med.

[r18] Gandini S, Boniol M, Haukka J, Byrnes G, Cox B (2011). Meta-analysis of observational studies of serum 25-hydroxyvitamin D levels and colorectal, breast and prostate cancer and colorectal adenoma.. Int J Cancer.

[r19] Garland FC, Garland CF, Gorham ED, Young JF (1990). Geographic variation in breast cancer mortality in the United States: a hypothesis involving exposure to solar radiation.. Prev Med.

[r20] Gorham ED, Garland CF, Garland FC (1989). Acid haze air pollution and breast and colon cancer mortality in 20 Canadian cities.. Can J Public Health.

[r21] Gorham ED, Garland FC, Garland CF (1990). Sunlight and breast cancer incidence in the USSR.. Int J Epidemiol.

[r22] Grant WB (2002). An estimate of premature cancer mortality in the U.S. due to inadequate doses of solar ultraviolet-B radiation.. Cancer.

[r23] Grant WB (2010). An ecological study of cancer incidence and mortality rates in France with respect to latitude, an index for vitamin D production.. Dermatoendocrinology.

[r24] Guyton KZ, Kensler TW, Posner GH (2003). Vitamin D and vitamin D analogs as cancer chemopreventive agents.. Nutr Rev.

[r25] Haddad JG, Chyu KJ (1971). Competitive protein-binding radioassay for 25-hydroxycholecalciferol.. J Clin Endocrinol Metab.

[r26] Hanwell HE, Vieth R, Cole DE, Scillitani A, Modoni S (2010). Sun exposure questionnaire predicts circulating 25-hydroxyvitamin D concentrations in Caucasian hospital workers in southern Italy.. J Steroid Biochem Mol Biol.

[r27] Holick MF (2007). Vitamin D deficiency.. N Engl J Med.

[r28] Hollis BW (2005). Circulating 25-hydroxyvitamin D levels indicative of vitamin D sufficiency: implications for establishing a new effective dietary intake recommendation for vitamin D.. J Nutr.

[r29] Huberman M, Langholz B (1999). Application of the missing-indicator method in matched case-control studies with incomplete data.. Am J Epidemiol.

[r30] John EM, Schwartz GG, Dreon DM, Koo J (1999). Vitamin D and breast cancer risk: the NHANES I Epidemiologic follow-up study, 1971–1975 to 1992. National Health and Nutrition Examination Survey.. Cancer Epidemiol Biomarkers Prev.

[r31] John EM, Schwartz GG, Koo J, Wang W, Ingles SA (2007). Sun exposure, vitamin D receptor gene polymorphisms, and breast cancer risk in a multiethnic population.. Am J Epidemiol.

[r32] Knight JA, Lesosky M, Barnett H, Raboud JM, Vieth R (2007). Vitamin D and reduced risk of breast cancer: a population-based case-control study.. Cancer Epidemiol Biomarkers Prev.

[r33] Kraft P, Cox D, Paynter R, Hunter D, De Vivo I (2005). Accounting for haplotype uncertainty in matched association studies: a comparison of simple and flexible techniques.. Genet Epidemiol.

[r34] Krishnan AV, Feldman D (2011). Mechanisms of the anti-cancer and anti-inflammatory actions of vitamin D.. Annu Rev Pharmacol Toxicol.

[r35] Kuper H, Yang L, Sandin S, Lof M, Adami HO (2009). Prospective study of solar exposure, dietary vitamin D intake, and risk of breast cancer among middle-aged women.. Cancer Epidemiol Biomarkers Prev.

[r36] Lin J, Manson JE, Lee IM, Cook NR, Buring JE (2007). Intakes of calcium and vitamin D and breast cancer risk in women.. Arch Intern Med.

[r37] Lowe LC, Guy M, Mansi JL, Peckitt C, Bliss J (2005). Plasma 25-hydroxy vitamin D concentrations, vitamin D receptor genotype and breast cancer risk in a UK Caucasian population.. Eur J Cancer.

[r38] Lowe L, Hansen CM, Senaratne S, Colston KW (2003). Mechanisms implicated in the growth regulatory effects of vitamin D compounds in breast cancer cells.. Recent Results Cancer Res.

[r39] McCullough ML, Rodriguez C, Diver WR, Feigelson HS, Stevens VL (2005). Dairy, calcium, and vitamin D intake and postmenopausal breast cancer risk in the Cancer Prevention Study II Nutrition Cohort.. Cancer Epidemiol Biomarkers Prev.

[r40] McCullough ML Stevens VL Diver WR Feigelson HS Rodriguez C 2007 Vitamin D pathway gene polymorphisms, diet, and risk of postmenopausal breast cancer: a nested case-control study. Breast Cancer Res 9 1 R9; 10.1186/bcr1642 17244366 PMC1851389

[r41] Millen AE, Pettinger M, Freudenheim JL, Langer RD, Rosenberg CA (2009). Incident invasive breast cancer, geographic location of residence, and reported average time spent outside.. Cancer Epidemiol Biomarkers Prev.

[r42] Miyamoto K, Kesterson RA, Yamamoto H, Taketani Y, Nishiwaki E (1997). Structural organization of the human vitamin D receptor chromosomal gene and its promoter.. Mol Endocrinol.

[r43] Mohr SB, Garland CF, Gorham ED, Grant WB, Garland FC (2008). Relationship between low ultraviolet B irradiance and higher breast cancer risk in 107 countries.. Breast J.

[r44] Nejentsev S, Godfrey L, Snook H, Rance H, Nutland S (2004). Comparative high-resolution analysis of linkage disequilibrium and tag single nucleotide polymorphisms between populations in the vitamin D receptor gene.. Hum Mol Genet.

[r45] Norval M, Wulf HC (2009). Does chronic sunscreen use reduce vitamin D production to insufficient levels?. Br J Dermatol.

[r46] Percy C, Van Holten V, Muir C, eds. (1990). International Classification of Diseases for Oncology. 2nd ed.

[r47] Pukkala E, Notkola V (1997). Cancer incidence among Finnish farmers, 1979–93.. Cancer Causes Control.

[r48] Rejnmark L, Lauridsen AL, Brot C, Vestergaard P, Heickendorff L (2006). Vitamin D and its binding protein Gc: long-term variability in peri- and postmenopausal women with and without hormone replacement therapy.. Scand J Clin Lab Invest.

[r49] Robien K, Cutler GJ, Lazovich D (2007). Vitamin D intake and breast cancer risk in postmenopausal women: the Iowa Women’s Health Study.. Cancer Causes Control.

[r50] Rosso S, Minarro R, Schraub S, Tumino R, Franceschi S (2002). Reproducibility of skin characteristic measurements and reported sun exposure history.. Int J Epidemiol.

[r51] Rothman K, Greenland S. (1998). Modern Epidemiology. 2nd ed.

[r52] Sinnwell JP, Schaid DJ. (2013). Statistical Analysis of Haplotypes with Traits and Covariates when Linkage Phase is Ambiguous.. http://cran.r-project.org/web/packages/haplo.stats/haplo.stats.pdf.

[r53] Sowers MR, Wallace RB, Hollis BW, Lemke JH (1986). Parameters related to 25-OH-D levels in a population-based study of women.. Am J Clin Nutr.

[r54] Thieden E, Philipsen PA, Heydenreich J, Wulf HC (2009). Vitamin D level in summer and winter related to measured UVR exposure and behavior.. Photochem Photobiol.

[r55] Welsh J, Wietzke JA, Zinser GM, Byrne B, Smith K (2003). Vitamin D-3 receptor as a target for breast cancer prevention.. J Nutr.

[r56] Welsh J, Wietzke JA, Zinser GM, Smyczek S, Romu S, et al. (2002). Impact of the Vitamin D3 receptor on growth-regulatory pathways in mammary gland and breast cancer.. J Steroid Biochem Mol Biol.

[r57] Wiklund K, Dich J (1994). Cancer risks among female farmers in Sweden.. Cancer Causes Control.

[r58] Yang L, Veierod MB, Lof M, Sandin S, Adami HO (2011). Prospective study of UV exposure and cancer incidence among Swedish women.. Cancer Epidemiol Biomarkers Prev.

[r59] Zinser GM, McEleney K, Welsh J (2003). Characterization of mammary tumor cell lines from wild type and vitamin D3 receptor knockout mice.. Mol Cell Endocrinol.

