# Supplementary material for: Sun Exposure, Vitamin D Receptor Genetic Variants, and Risk of Breast Cancer in the Agricultural Health Study
Source: Environ Health Perspect. 2013 Nov 19;122(2):165–71. doi: 10.1289/ehp.1206274 (PMC3915256; doi:10.1289/ehp.1206274)
Supplement: (295 kB) PDF [file ehp.1206274.s001.508.pdf]

Environmental Health Perspectives doi:10.1289/ehp.1206274

Supplemental Material
Sun Exposure, Vitamin D Receptor Genetic Variants, and Risk of Breast Cancer in the Agricultural Health Study

Lawrence S. Engel, Jaya Satagopan, Camelia S. Sima, Irene Orlow, Urvi Mujumdar, Joseph Coble, Pampa Roy, Sarah Yoo, Dale P. Sandler, and Michael C. Alavanja

Table of Contents

Supplemental Material, Table S1……..…………………………………………………….2

Supplemental Material, Table S2………………………………………….……………….4

Supplemental Material, Table S1. Selected characteristics at enrollment of breast cancer cases and controls in the nested case-control study.

Characteristic	Cases (n = 293)[a]		Controls (n = 586)[a]		Adjusted OR[b]	95% CI
	No.[c]	%	No.[c]	%		
Age (years)						
18-39	30	10.2	69	11.8	NA	
40-49	67	22.9	142	24.2	NA	
50-59	122	41.6	234	39.9	NA	
60-69	60	20.5	119	20.3	NA	
70-86	14	4.8	22	3.8	NA	
Race						
White, Hispanic and non-Hispanic	281	97.9	574	98.0	NA	
Other	6	2.1	12	2.0	NA	
Missing	6		0			
State of residence						
Iowa	196	66.9	392	66.9	NA	
N. Carolina	97	33.1	194	33.1	NA	
Highest educational level						
< high school	10	3.9	33	6.3	0.5	(0.2, 1.1)
High school	107	41.3	229	43.4	0.9	(0.6, 1.2)
> high school	142	54.8	266	50.4	1	Ref
Missing	34		58			
Smoking						
Never	207	74.5	417	73.7	1	Ref
Former	59	21.2	110	19.4	1.8	(0.9, 3.6)
Current	12	4.3	39	6.9	2.0	(0.9, 4.2)
Missing	15		20			
First degree family history of breast cancer						
Yes	62	22.1	81	14.2	1.8	(1.2, 2.6)
No	219		491		1	
Missing	12		14			
BMI (kg/m^2)						
< 25.0	111	47.4	190	41.5	1	Ref
25.0-29.9	77	32.9	170	37.1	0.8	(0.5, 1.1)
≥ 30.0	46	19.7	98	21.4	0.8	(0.5, 1.3)
Missing	59		128			
Age at menarche (years)						
< 12	27	12.2	59	14.6	1	Ref
12-14	178	80.2	307	75.8	1.2	(0.7, 2.0)
≥ 15	17	7.7	39	9.6	0.9	(0.4, 1.8)
Missing	71		181			
Parity						

Characteristic	Cases (n = 293)[a] No.[c]	Cases %	Controls (n = 586)[a] No.[c]	Controls %	Adjusted OR[b]	95% CI
Nulliparous	4	1.4	7	1.2	0.9	(0.2, 3.3)
1	29	10.2	45	7.7	1.3	(0.8, 2.2)
≥ 2	243	88.4	506	91.1	1	Ref
Missing	17		28			
Age at first birth (years)[d]						
≤ 20	47	22.7	88	23.7	1	Ref
20-30	137	66.2	263	70.9	1.0	(0.8, 1.2)
> 30	23	11.1	20	5.4	1.7	(1.2, 2.6)
Missing	65		180			
Menopausal status						
Post-menopausal	187	67.8	383	68.1	0.9	(0.6, 1.6)
Pre-menopausal	89		179		1	
Missing	17		24			
Age at menopause (years)[e]						
< 45	65	34.9	138	36.5	1	Ref
45-49	42	22.6	92	24.3	0.8	(0.5, 1.3)
50-54	64	34.4	110	29.1	1.2	(0.7, 1.8)
≥ 55	15	8.1	38	10.1	1.2	(0.7, 2.3)
Missing	1		5			
Usual sunblock use at enrollment						
Yes	137	46.8	263	44.9		
No	156	53.2	323	55.1		
Tumor estrogen receptor status						
ER+	181	76.6	N/A			
ER-	55	23.4	N/A			
Missing	57					
Tumor progesterone receptor status						
PR+	161	68.8	N/A			
PR-	73	31.2	N/A			
Missing	59					

[a]Controls were randomly selected with replacement and include 19 subjects who were each selected as controls for two cases and four subjects who were each selected as both a control and, at a later time, a case. [b]ORs were estimated using conditional logistic regression, with all factors adjusted for the other factors in the table, except where indicated and except for matching factors—age at enrollment (5-year age groups), enrollment period (1993-1995, 1996-1997), race (white, other), and state of residence (Iowa, North Carolina)—and with no imputed data. [c]Number of cases or controls indicated for some factors may be less than total number of cases or controls due to missing data. [d]Restricted to parous women. [e]Restricted to post-menopausal women.

Supplemental Material, Table S2. Most common 50% of haplotypes in *VDR* Blocks B and C (from among 123 in Block B and 178 in Block C) in the nested case-control study.

**Most common haplotypes**[a]
Block B[b]
B1: G T C A C T C C C T A
B2: G T C A C T T A C T A
B3: G T C A T T T A C T G
B4: G T C A T T T C C T A
B5: T C A G C T T A C T A
B6: T C A G C T T C G C A
Block C[b]
C1: C C C A C G C G C C C C C
C2: C C G T C A T G C C A G C
C3: C T G T G A C G C C A G C
C4: T C C T C G C G C C C C C
C5: T C G T C G C G C T C C A
C6: T T G T G A C G C C A G C
C7: T T G T G A C G C C C G A

[a]Additional detail available in Engel et al. (2012). [b]Blocks based on Nejentsev et al. (2004), with the order of SNPs as listed – Block B: rs739837, rs731236, rs7975232, rs2239182, rs2107301, rs2239181, rs2238139, rs2189480, rs3782905, rs7974708, rs11168275; Block C: rs2408876, rs1989969, rs2238135, rs10875694, rs3922882, rs11168287, rs7299460, rs11168314, rs4073729, rs3923693, rs4760674, rs6823, rs2071358.
